# Exosomal circRNAs as novel cancer biomarkers: Challenges and opportunities

**DOI:** 10.7150/ijbs.48782

**Published:** 2021-01-14

**Authors:** Shuai Wang, Yanhan Dong, Anjing Gong, Huimin Kong, Jinning Gao, Xiaodan Hao, Yongmei Liu, Zibo Wang, Yuqiao Fan, Chengyu Liu, Wenhua Xu

**Affiliations:** 1 Department of Inspection, The medical faculty of Qingdao University, Qingdao 266003, China.; 2 Institute of Translational Medicine, Qingdao University, Qingdao, 266003, China.; 3 Department of Neurosurgery, The affiliated hospital of Qingdao University, Qingdao 266003, China.; 4 School Hospital, Shandong University of Science and Technology, Qingdao 266003, China.

**Keywords:** CircRNAs, exosomes, exosomal circRNAs, liquid biopsy, biomarkers

## Abstract

Identifying high specificity and sensitivity biomarkers has always been the focus of research in the field of non-invasive cancer diagnosis. Exosomes are extracellular vesicles with a lipid bilayer membrane that can be released by all types of cells, which contain a variety of proteins, lipids, and a variety of non-coding RNAs. Increasing research has shown that the lipid bilayer can effectively protect the nucleic acid in exosomes. In cancers, tumor cell-derived exosomal circRNAs can act on target cells or organs through the transport of exosomes, and then participate in the regulation of tumor development and metastasis. Since exosomes exist in various body fluids and circRNAs in exosomes exhibit high stability, exosomal circRNAs have the potential as biomarkers for early and minimally invasive cancer diagnosis and prognosis judgment. In this review, we summarized circRNAs and their biological roles in cancers, with the emerging value biomarkers in cancer diagnosis, disease judgment, and prognosis observation. In addition, we briefly compared the advantages of exosomal circRNAs as biomarkers and the current obstacles in the exosome isolation technology, shed light to the future development of this technology.

## Introduction

Cancer is a serious disease that threatens human health. Given that its onset is insidious and the early symptoms are not obvious, at the time of clinical diagnosis, many patients are already in the advanced stage of the disease 1. Early detection, early diagnosis, and early treatment are crucial to improve the five-year survival rates of patients with cancer. Compared with traditional histological biopsy, the liquid biopsy technique developed in recent years to detect circulating tumor markers in body fluids has attracted increasing attention in early diagnosis of tumors and detection of therapeutic effects. The main advantages of liquid biopsy include non-invasiveness, simplicity, and speed. However, there are still few mature biomarkers available to guide clinical practice, so it is urgent to investigate novel stable and reliable tumor biomarkers in cancer research.

Exosomes are one of the main detection materials for liquid biopsy, and disease-specific proteins and nucleic acids in exosomes are also the focus of research in this field. Circular RNAs (circRNAs) are a type of RNAs that are more abundant, specific, and highly organized, compared to other types of RNAs. CircRNAs play an important endogenous regulatory role in many human diseases, and recent studies have revealed that circRNAs can stably exist in peripheral blood, saliva, urine, gastric juice, seminal plasma and other body fluids 2-9. At present, the detection of a specific circRNA in fluid exosomes of cancer patients provides new ideas and research methods for liquid biopsy of tumors 9. It is expected that liquid biopsy will allow non-invasive examination of cancer, and will be particularly significant for deep tissue lesions which it is difficult to obtain in the clinic.

## Overview of Exosomes

Exosomes are vesicles with a bilayer membrane structure formed by a series of regulatory processes, such as “endocytosis-fusion-exclusion” of most cells (including T cells, platelets, and cancer cells), with a diameter of about 50-150 nm 10. Exosomes were first discovered as extracellular vesicles during the maturation of reticulocytes 11. It was then found that almost all types of cells can normally secrete extracellular vesicles, which play an important role in the exchange of information between cells. Indeed, exosomes are widely present in blood, urine, saliva, bile, cerebrospinal fluid, and other common body fluids 10, containing a variety of proteins, signal lipids, and various types of nucleic acid molecules. In addition, the lipid bilayer structure of exosomes can effectively prevent its contents from degradation and transport it to specific target cells 12. For example, tumor-derived exosomes can carry tumor-related nucleic acids and proteins, and when delivered to the target cells, they can contribute to immune regulation, mediate angiogenesis and regulate the microenvironment of tumor metastasis.

As exosomes have good secretory characteristics and stable vesicular structure, circulating exosomes in biological fluid may be applied as indirect representatives of exosomal components in the microenvironment of tumor tissues. Therefore, exosomal components can be used as biomarkers for tumor diagnosis and observation of treatment. Earlier studies found that the expression level of miRNAs in fluid exosomes during tumor progression were significantly different from those in healthy people 13. For example, Huang et al. evaluated miRNAs of plasma exosomes in patients of prostate cancer and indicated the potential of miR-1290/miR-375 in exosomes as a biomarker for diagnosis and prognostic evaluation of prostate cancer 9. In recent years, with further exploring in the field of molecular biology, researchers have found that apart from miRNA, other nucleic acid molecules in exosomes may also play an emerging role in tumor detection and diagnosis 14.

## General characteristics of CircRNAs and their roles in Cancer

Understanding the formation mechanism and biological characteristics of a new molecule is essential for exploring its function, regulation and application value in diseases. Here, we first summarized the biological formation, biological functions of circRNA and its regulatory role in cancer. CircRNA is a novel non-coding RNA molecule that is distinguished from the traditional linear RNA with a covalently closed loop 14. It was first discovered in RNA viruses in 1976 15. Due to technological limitations at that time, circRNA was only regarded as a class of low-abundance RNAs that were incorrectly spliced by exon transcripts. Therefore, researchers did not pay much attention to circRNAs. Recently, with widespread use of high-throughput sequencing technology and rapid development of bioinformatics, circRNA has been found to be stable, abundant, and ubiquitous in mammals. CircRNA has been found extensively in different tissues or the same tissue at different statuses, with specific cell phenotypes and developmental stages 16. Mainly due to the lack of 5'-end cap structure and 3' poly-A tail structure, circRNAs can resist the hydrolysis of RNA exonuclease. Therefore, their half-life can reach up to 48 hours, with higher stability than linear RNA with a half-life of 10 hours 17.

### Biological formation of CircRNAs

**Figure 1:**
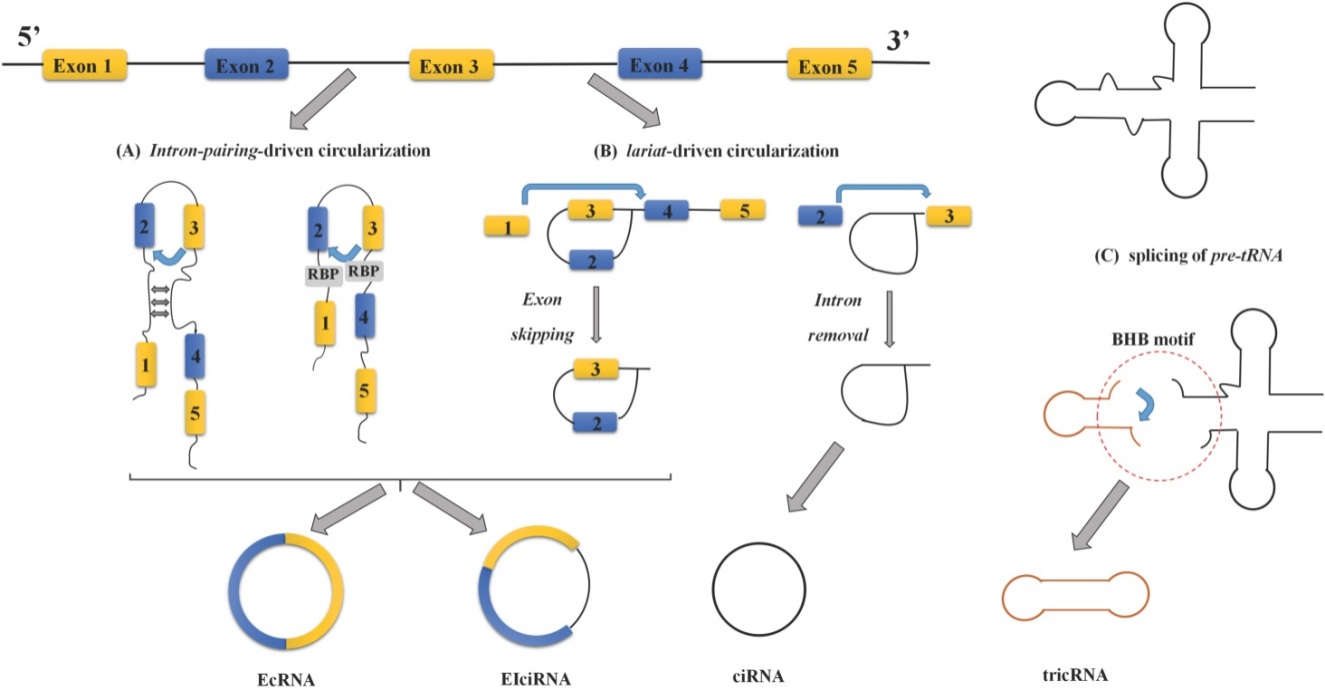
** The formation process and classification of circRNA. (A)** Intron-pairing-driven circularization:The formation of the loop structure occurs through the pairing of flanking introns and reverse complementary sequences or the action of RBPs, and then removing or retaining the introns and connecting the exons to form EcRNAs or EIciRNAs, respectively. **(B)** Lariat-driven circularization: This process is associated with exon skipping or intron removal, in which one or more exons of the transcript are skipped, and it contributes to an exon-containing lariat. The formed lasso intermediate produces circRNA by further splicing. **(C)** Splicing of pre-tRNA: CircRNA derived from pre-tRNA.

Based on the different original sequences, circRNAs are mainly divided into three categories: exon-intron circRNAs (EIciRNAs), exonic circRNAs (EcRNAs), and intron-derived circRNAs 8, 17-19. The latter mainly include circular intronic RNAs (ciRNAs) derived from pre-mRNA and tRNA intronic circular RNAs (tricRNAs) 18-20. Most circRNAs are derived from gene exons, but some of them also harbor introns, which are formed by backsplicing instead of classical splicing 21. Researchers have proposed two different models of exon/intron circularization: intron-pairing-driven circularization and lariat-driven circularization 17, 22. The intron-pairing-driven circularization model believes that the loop structure can be formed by base pairing or RBPs between the complementary sequences on both sides of the circular exon, while the intron sequence is removed or retained in the loop structure to generate EcRNAs or EIciRNAs, respectively 8, 23, 24 (Figure 1A). The complementary sequence in the intron flanking the exon is an indispensable cis-acting element for circRNA biogenesis 25. Because such base pairing forms a hairpin structure, it leads to the downstream 5' splice site and the upstream 3' splice site close enough for circularization. The lariat-driven circularization model is associated with exon-skipping events or during intron removal from pre-mRNAs 8, 24 (Figure 1B). In exon-skipping events, one or more exons of the transcript are skipped, therefore contributing to an exon-containing lariat. The lariat intermediate is further spliced to generate an EcRNA 26. In another mechanism, the processing of the intronic lariat relies on the 7-nt GU rich sequence near the 5' splice site and the 11-nt C rich sequence near the branch site. Immediately afterward, the 3' tail downstream branch point site is removed to form a stable ciRNA 8, 18, 24.

Most circRNAs currently studied were derived from pre-mRNA, and a small portion of intron-derived circRNAs were derived from pre-tRNA. In the pre-tRNA maturation process, the tRNA splicing endonuclease (TSEN) compound cleaves the pre-tRNA containing introns at the typical bulge-helix-bump (BHB) motif. The resulting intron ends are ligated by RtcB ligase to form a stable circRNA, called tricRNA 19, 20 (Figure 1C).

### Biological function of CircRNA

As the research tools and methods for studying circRNA continue to improve in terms of quantity and quality, the researchers here summarized the main functions of circRNA.

### microRNA sponge

There are microRNA binding sites on circRNA, and circRNA can regulate downstream gene expression by binding to microRNA, thereby exerting corresponding biological functions 27. A large number of studies have found that a variety of circRNAs have microRNA sponge functions, such as CDR1as, circPVT1 and so on 28, 29.

### Interacting with RNA binding proteins

CircRNA can bind to certain functional proteins to perform biological functions. A typical example is that the RNA cleavage factor muscleblind-like (MBL) can promote the circularization of circular RNA Mbl (circMbl), and circMbl can also bind to MBL to reduce the concentration of MBL, thereby reducing the synthesis of circMbl 30.

### Regulating parental gene expression

Since circRNA can be regarded as a splicing isoform, it may be translated into a functional protein or reduce the normalized splicing transcript that can be translated into a functional protein, thereby regulating gene transcription at a variable regulatory level 31.

### Coding protein

Current studies have revealed that circRNA can also be directly translated into protein or polypeptide to perform its biological function 32. For example, in the 1980s, Kos et al. discovered that single-stranded negative-strand circRNA at the core of hepatitis D could be transcribed and translated into hepatitis D core antigen 32.

### CircRNAs in Cancer

#### CircRNA and tumor development

The regulatory role of circRNAs in various tumors has been in the focus of molecular oncology research recently. The latest study has found that circUHRF1, which is highly expressed in oral squamous cell carcinoma cell lines, can be bound to miR-526b-5p via sponging and positively regulating c-Myc, while c-Myc can promote the expression of TGF-β1 and ESRP1 33. This process plays a key role in promoting cancer, and ESRP1 can promote the generation of circUHRF1 by combining the flanking introns of circUHRF1 to finally form a positive feedback regulatory loop 33. At the same time, researchers found that circTLK1, which is highly expressed in kidney cancer, can promote the progression of renal cancer through the miR-136-5p/CBX4 pathway 34. Moreover, the expression level of circTLK1 in clinical samples was positively correlated with cancer cell metastasis and poor prognosis, and it has the potential as a diagnostic molecule and therapeutic target for kidney cancer 34. Furthermore, Gu et al. found that circGPRC5a, which is highly expressed in bladder cancer tissues and bladder cancer stem cells (CSCs), could promote the metastasis of bladder cancer by translating the polypeptide and binding the polypeptide to GPRC5a 35. The studies indicate that circRNAs can participate in the occurrence and development of tumors through various targets or different signal pathways.

#### CircRNAs as tumor biomarkers

Based on the relationship between circRNA and tumor development, researchers gradually confirmed the diagnostic value of circRNA in tumors by comparing the differential expression of circRNA in body fluids of cancer patients and healthy controls. In an earlier study, Zhang et al found a significantly reduced expression of has_circ_0001445 in plasma of HCC patients than those in hepatitis B, cirrhosis, and healthy controls 36. Moreover, the results of the receiver operating characteristic curve (ROC) analysis showed that plasma has_circ_0001445 could be used as a more accurate marker to distinguish HCC cases from healthy people, cirrhosis, or hepatitis B patients 36. Similar studies also performed by detecting low expression of has_circ_0000190 detected in the serum of gastric cancer by Chen et al. Its expression was related to tumor size, lymph node metastasis, distant metastasis, TNM stage and CA19-9 level. And its diagnostic sensitivity and specificity are superior to traditional markers such as CEA and CA19-9 37. In the latest research, Lu et al. identified that the expression of has_circ_0006848 in plasma was significantly inhibited in patients with early gastric cancer compared with healthy volunteers 14. Moreover, the area under the ROC curve (AUC) of plasma has_circ_0006848 was 0.733, indicating a superior diagnostic value. The plasma hsa_circ_0006848 was combined with the CEA, CA19-9, and CA72-4 levels increased the AUC to 0.825 14.

Furthermore, differential expression of circRNA in body fluids also provides an emerging prospect as tumor biomarkers. Saliva has been increasingly considered as an ideal body fluid specimen for disease research due to several advantages, such as convenient material extraction, non-invasive process, rapid detection, and low cost. A growing number of studies have explored circRNA in saliva as a marker of disease due to its high stability. Zhao et al. firstly identified the circRNA expression profiles in saliva of three oral squamous cell carcinoma patients and three healthy controls by microarray analysis 4. Then, the three most prominent circular RNAs (has_circ_0001874, has_circ_0001971, and has_circ_0008068) and the three most significant down-regulations (has_circ_0000140, has_circ_0002632 and has_circ_0008792) were selected for differential expression of circRNA for further verification 4. The differential expression of circRNA had a diagnostic value for oral squamous cell carcinoma, in which the AUC value of has_circ_0001874 was the highest; the diagnostic accuracy of the combined diagnosis of has_circ_0001874 and has_circ_0001971 was additionally enhanced 4. Furthermore, it was reported that the expression level of circRNAs in saliva of these patients of 1 month after surgery were significantly lower than those of 1 month before surgery 4. Their study confirmed the importance of salivary circRNA in the preoperative diagnosis and prognosis judgment of oral squamous cell carcinoma. It also laid the foundation for the application of humoral circRNA as a biomarker for human diseases.

The functions of circRNA biomarkers in body fluids of various tumors were listed in Table 1.

## Exosomal CircRNAs in Cancer

In recent years, with the rise of liquid biopsy technology and exosome research, some researchers have investigated the link between exosomes and circRNAs in tumors by sequencing the tumor-derived exosomal circRNA. They concluded that circRNA in exosomes can work as a biomarker for tumor diagnosis, thus providing a new development direction for tumor diagnosis 9.

**Figure 2:**
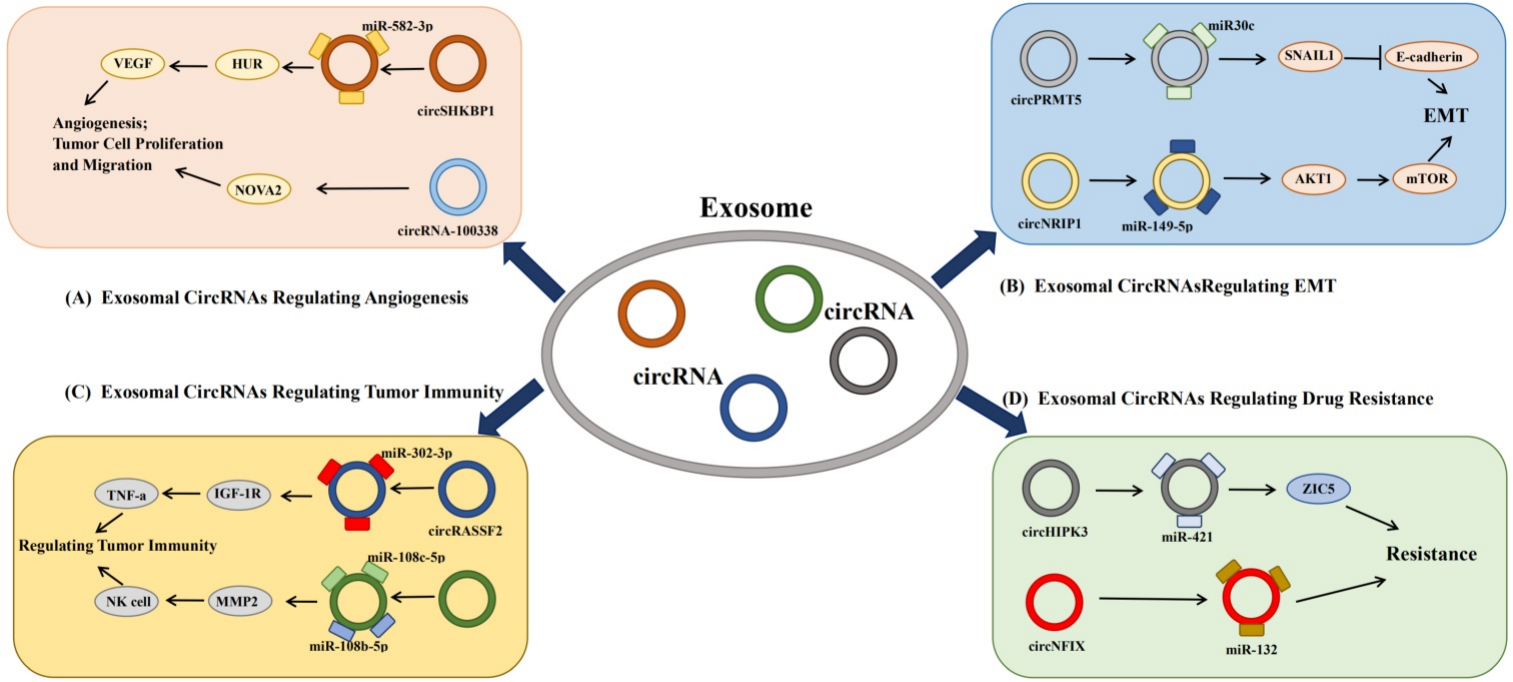
** The regulatory role of exosomal circRNA in tumors.** Donor cells secrete exosomal circRNA into recipient cells, which can affect the tumor microenvironment and tumor growth and metastasis by competitively binding miRNA and acting on downstream target genes. **(A)** Angiogenesis: Exosomal circRNAs can regulate tumor angiogenesis and permeability of vascular endothelial cells through miRNA sponge action or direct binding to proteins. **(B)** EMT: Exosomal circRNAs can enter different tumor cells and participate in the regulation of EMT and invasive growth of tumor cells. **(C)** Regulating tumor immunity: Exosomal circRNA can participate in the regulation of tumor immune microenvironment through multiple channels. **(D)** Regulating drug resistance: Exosomal circRNA participates in the regulation of drug resistance of multiple chemotherapeutics through multiple channels.

Exosomes are important mediators of metastasis and angiogenesis in the tumor microenvironment. They mediate the communication between tumor cells and mesenchymal cells and affect tumor progression 38, 39. A large number of studies have found that exosomes derived from tumor cells can enter the body fluid circulation by carrying tumor-specific circRNA and participating in the regulation of tumor development and metastasis. We first briefly summarized their regulatory roles in the occurrence and development of cancer (Figure 2), so as to more easily understand their value and significance.

### The regulation of exosomal CircRNAs on angiogenesis

Active tumor angiogenesis is the main reason for accelerated tumor proliferation, early metastasis and poor prognosis. Exosomal circRNAs can induce the formation of vascular cavity and increase the permeability of vascular endothelium by regulating the communication between tumor cells. They further contribute to the formation of tumor hypoxic microenvironment and promote the formation of malignant phenotype of tumor cells 40.

For instance, exosomes circRNA-100338 interact with NOVA2 to regulate the angiogenesis and vascular permeability of human umbilical vein endothelial cells (HUVECs), thereby affecting the migration ability of liver cancer cells and promoting HCC metastasis 40 (Figure 2A). *In vivo* knockdown of exosomes circRNA-100338 significantly inhibits tumor growth and reduces lung metastatic nodules 40. In addition, vascular endothelial growth factor (VEGF) is one of the important factors that promote the proliferation of endothelial cells. Xie et al. found that exosomal circSHKBP1 can promote the proliferation, migration, invasion and angiogenesis of gastric cancer cells by regulating the miR-582-3p/HUR/VEGF pathway 41 (Figure 2A).

### Exosomal CircRNAs' Regulation of EMT

Epithelial-mesenchymal transition (EMT) is a reversible dedifferentiation process. In this process, the tumor microenvironment is remodeled; meanwhile, epithelial cells may lose polarity, and obtain a mesenchymal phenotype. EMT is considered to be the central mechanism of tumor invasion and metastasis.

Exosomal circPRMT5 is secreted into the tumor microenvironment and regulates the SNAIL1/E-cadherin signaling pathway by competing with miR30c, up-regulating SNAIL1 and inhibiting E-cadherin; thereby promoting the EMT of urothelial carcinoma of the bladder (UCB) cells, affecting the tumor microenvironment, and facilitating tumor metastasis 5 (Figure 2B). Similarly, Zhang et al. revealed that exosomal circNRIP1 can act as a sponge for miR-149-5p, further modulating the AKT1/mTOR axis to increase EMT, change tumor metabolism homeostasis, and eventually promote tumor metastasis 42 (Figure 2B). In summary, those studies showed that exosomal circRNAs play an important role in tumorigenesis and development by regulating EMT.

### Exosomal CircRNA regulating tumor immunity

Tian et al. found that exosomal circRASSF2 can competitively bind miR302b-3p, accelerate the expression of IGF-1R, and then regulate the growth and metastasis of laryngeal cancer 43 (Figure 2C). Prior to this, studies have reported that in breast cancer epithelial cells, inhibition of IGF-1R increases cell stress, CC motif chemokine ligand 2 (CCL2), IL-10, and IL-6, and reduces TNF-α 44. Thus, through the infiltration of CD11b^+^ and CD45^+^ monocytes, the expression of MMP2, MMP3, and MMP9, and matrix remodeling promote the formation of aggressive tumor microenvironment 44. Therefore, exosomal circRNAs may also affect tumor immune microenvironment and promote tumor progression by regulating IGF-1R. Circ-0008433 regulates the expression of matrix metallopeptidase 2 (MMP2) by competitively binding to miR-181c-5p and miR-181b-5p, which further recruits NK cells to attack arterial elastic fibers, remodel blood vessels, and promote the progression of aneurysms 45 (Figure 2C).

### Role of exosomal CircRNAs in regulation of drug resistance

Chemotherapy resistance is one of the main obstacles to tumor treatment. Exosomes can deliver multi-drug resistance proteins (MDR) and circRNAs to recipient cells, and they have the ability to regulate chemotherapy resistance. Temozolomide (TMZ) is a commonly used chemotherapy drug for the treatment of glioma 46. Ding et al. found that exosomal circNFIX can promote tumor growth 47. Knockdown of exosomes circNFIX enhanced the sensitivity of glioma cells to TMZ, in which the specific mechanism also involves the interaction of exosomes circNFIX with miR-132 47 (Figure 2D). This suggests that exosomal circNFIX may be a new target for the treatment of TMZ-resistant glioma. Similarly, Han et al. found that exosomal circHIPK3 can facilitate the progression of glioma cells and the resistance of glioma cells to TMZ by regulating the miR-421/ZIC5 axis 48. Zhang et al. found that knock out circUHRF1 in HCC cells improved the sensitivity of anti-PD-1 treatment and improved the overall survival rate of patients 40 (Figure 2D), suggesting that exosomal circUHRF1 may lead to resistance to anti-PD-1 immunotherapy and provide a potential treatment strategy for patients with HCC. The above studies imply that a variety of exosomal circRNAs are closely related to tumor resistance, and they may become novel targets for the treatment of tumor resistance.

## Exosomal CircRNAs as Cancer biomarkers

After elucidating the regulatory role of exosomal circRNAs in cancer, we will introduce their value as cancer markers, and preliminarily summarize the advantages and disadvantages of exosomal circRNAs as cancer markers and the possible technology problems.

In exploring the functions of exosomal circRNAs, researchers found that circRNA in blood was mainly present in blood exosomes and transported by exosomes 9. More than 1,000 circRNAs were found in human serum exosomes, which is six times greater than linear RNA, suggesting that circRNAs are more likely to be enriched in exosomes than linear RNA 9. For example, cells contained a large amount of ciRS-7 mRNA, but the expression of ciRS-7 in exosomes was significantly higher than the corresponding mRNAs level, and the expression level of mRNA in exosomes is low 49. This suggests that ciRS-7 is easier to sort into exosomes than mRNA. After serum was cultured at room temperature for 24 hours, the level of exosomal circRNA remained stable, indicating that the stability of circRNA may be related to the protective effect of the double lipid membrane structure of exosomes.

At different stages of different development diseases, disease-related circRNA can be sorted into exosomes to be enriched and transported to target cells or target organs for release. The sorting of circRNAs into exosomes may be regulated by the following mechanisms: (1) Regulation of miRNAs. For example, Li et al. found that exosomal circCDR1as can act as miR-7 sponges. When miR-7 was ectopically expressed in liver cancer cells, the expression level of circCDR1as in exosomes was significantly down-regulated, while the expression of circCDR1as in cells increases 9. (2) lncRNA competitively participates in the sorting of circRNAs into exosomes. For example, Barbagallo et al. found that knock down of lncRNA UCA1 in serum exosomes blocked the mitogen-activated protein kinase (MAPK) signaling pathway, and up-regulated the expression of circHIPK3 29. (3) RNA binding proteins (RBPs) recognize RNAs with specific binding sequences to regulate the sorting of exosomes circRNAs 50, 51. The exosomes secreted by DKs-8 cells are enriched in RBPs, and these RBPs are involved in regulating the sorting process of circRNAs by binding to circFAT1 52.

### The potential of exosomal CircRNAs as tumor biomarkers

Exosomes exist in various body fluids, which is convenient for non-invasive detection 53. CircRNAs are stable, conservative, and specific expression of cells and tissues, which suggests that they have the potential to be used as molecular diagnostic and prognostic markers 54, 55. Exosomal circRNAs combine the advantages of exosomes will supply good application prospects for early non-invasive detection of biomarkers. Exosomes derived from pathological cells can carry their disease-specific circRNA into the peripheral blood. Therefore, the detection of exosomal circRNA in serum may be feasible in the diagnosis of tumor disease.

Many studies have shown that differential expression of exosomal circRNAs in the body fluid was associated with the pathological characteristics of tumor vascular invasion and TNM stage. Li et al. reported that the expression of circ-IARS was significantly increased in serum exosomes of patients with metastatic pancreatic cancer, and it was associated with tumor vascular invasion, liver metastasis, and TNM stage 56. Survival analysis showed that the expression of circ-IARS negatively correlated with the survival time of patients with pancreatic cancer, suggesting that circ-IARS in serum exosomes may be a non-invasive biomarker for early diagnosis and prognosis of pancreatic cancer 56. In addition, Li et al. found that the expression of exosomal circPDE8A in plasma was related to the progression and prognosis of PDAC patients, suggesting that exosomal circPDE8A may be an important indicator for early diagnosis and prognosis of PDAC 36.

There are many similar studies in other cancer models, and here we list the most representative ones. By comparing the exosomal circRNA in the serum of patients with stage III endometrial cancer and healthy controls, Xu et al. found that the expression levels of has_circ_0109046 and has_circ_0002577 in the serum exosomes of patients with endometrial cancer increased dramatically 57, indicating their potential as blood diagnostic markers for endometrial cancer. In the last two years of research, Lu et al. found that exosomal circ-RanGAP1 can promote the metastasis and development of gastric cancer by targeting the miR-877-3p/VEGFA axis 58. Circ-RanGAP1 was significantly up-regulated in both human and gastric cancer tissues, and its high expression level was closely related to TNM staging, lymph node metastasis, and poor survival 58. This suggests that circ-RanGAP1 has the potential to be a marker for preoperative diagnosis and prognostic monitoring of gastric cancer. At the same time, circPTGR1, which is highly expressed in highly metastatic HCC cells and in serum exosomes of HCC patients, is also related to clinical stage and prognosis 59. This indicates that circPTGR1 also has a certain potential as a biomarker in the preoperative staging and prognostic observation of HCC patients. A recent study has found that the continued high expression of exosomal circRNA-100338 in the serum of HCC patients undergoing therapeutic hepatectomy may be related to lung metastasis and poor survival 60. Hence, circRNA-100338 may become an indicator of poor prognosis.

In addition, in the study of laryngeal squamous cell carcinoma (LSCC), Tian et al. found that circRASSF2 can promote the progression of LSCC 43. In clinical samples, compared with healthy controls, the circRASSF2 level in serum exosomes of LSCC patients was significantly induced, which suggests that circRASSF2 also has a certain preoperative diagnostic value in LSCC 43. Similar studies have found that has_circ_0010522 can promote white fat browning, and that its expression level in gastric cancer plasma is positively correlated with white fat browning 61. This circRNA may play a certain auxiliary diagnostic value in the diagnosis of gastric cancer patients. Wu et al. found that exosomal circFNDC3B can act as a sponge for miR-1178 and promote the progress of papillary thyroid cancer (PTC) 8. Further experiments found higher prevalence of the exosomal circFNDC3B in the serum of PTC patients than in healthy individuals 8. Thus, the circRNA may be a new clinical molecular marker of PTC. In addition, Li et al. found that expression of exosomal circFECR1 was abnormally high in small cell lung cancer (SCLC) patients' serum, and it was linked with the clinical response to chemotherapy and lower survival rate 62. After the first-line chemotherapy of SCLC patients with partial or complete remission, the serum levels of exosomal circFECR1 were significantly reduced, which suggests that exosomal circFECR1 dynamically changed in parallel with the response to chemotherapy drugs 62. Therefore, exosomal circFECR1 may be a useful clinical indicator that can dynamically predict chemotherapy response. Compared with lung adenocarcinoma tissue without lymph node metastasis, exosomal circRNA-0056616 was more actived in lung adenocarcinoma tissue with lymph node metastasis 63. Moreover, the expression in the serum of SCLC patients with lymph node metastasis was significantly higher than those patients without lymph node metastasis 63. This implys that exosomal circRNA-0056616 may be a potential biomarker for prediction of lymph node metastasis in lung adenocarcinoma.

Exosomal circRNA detected in other body fluids also exhibits a certain diagnostic value. For example, abnormal metabolites of many diseases can be detected in urine. Due to easy retention and low detection cost, the urine samples are widely used in clinical practice. In serum or urine exosomes of patients with bladder urothelial cancer, the expression of circPRMT5 was significantly increased compared to that in healthy controls, and its expression level was related to tumor lymph node metastasis and progression 5. This shows that circPRMT5 in urine exosomes has a certain value in the diagnosis of bladder urothelial carcinoma.

Exosomal circRNAs in saliva as cancer biomarkers remains unclear. However, recent studies have implied the potential of GOLM1-NAA35 chimeric RNA (seG-NchiRNA) in salivary exosomes as a biomarker for early detection, efficacy evaluation, and relapse monitoring in esophageal squamous cell carcinoma 64. It is believed that with the improvement in theory and the advancement of technology, increasing potential of circRNAs in humoral exosomes as tumor biomarkers will be discovered.

### The advantages and disadvantages of exosomal CircRNAs as tumor markers

Although the current research on circRNAs in fluid exosomes is still in its infancy, many studies have shown their potential as cancer markers (Table 2). In order to objectively evaluate its feasibility, we briefly summarized the advantages and disadvantages of exosomal circRNAs as cancer markers.

**Figure 3:**
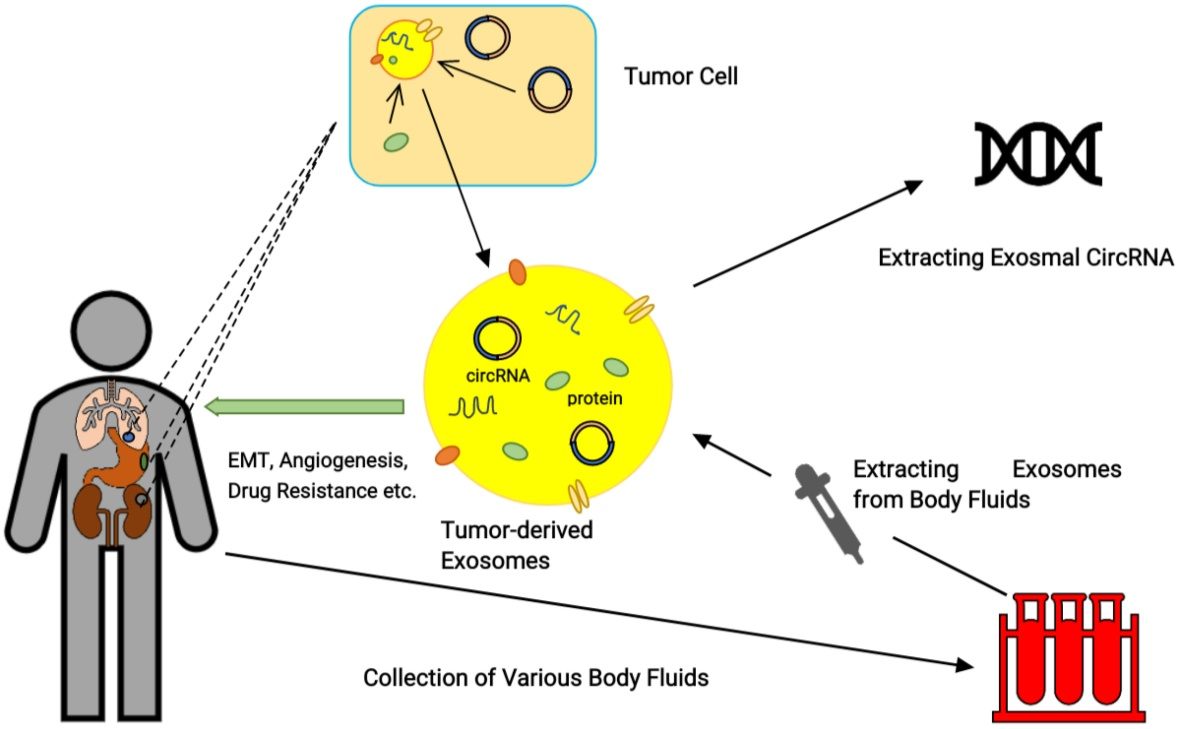
** Detection of cancer-related exosomal circRNA.** Tumor-derived exosomes contain a large number of tumor-specific nucleic acids and proteins. Exosomes function as an information carrier; by detecting the differentially expressed circRNAs in exosomes released into the body fluids, they can be important for the diagnosis of tumors. Rather than extracting circRNA directly from body fluids, extracting exosomes first and then extracting circRNA will result in a higher abundance and more differential expression of circRNA.

Firstly, as extracellular vesicles can be secreted by a variety of cells, the size of the exosomes and the heterogeneity of their content reflect the state and type of the originating cell. The contents of exosomes can be used as biomarkers for diagnosis or prognosis observation in various diseases 65. Secondly, the detection of exosome non-coding RNA has attracted increasing attention in the research of tumor diagnosis and treatment. As mentioned above, it is easier to sort circRNA into exosomes than linear RNAs such as miRNAs and lncRNAs 9. In addition, exosomes derived from cancers contain highly specific RNA, and they can also prevent the nucleic acid molecules from degradation by RNase in the blood 66 (Figure 3). Therefore, analysis based on the exosome-encapsulated non-coding RNA may be superior to whole plasma/serum analysis. Through animal models of kidney disease, some researchers have shown that the detection of exosomal miRNAs cannot be replaced by the detection of miRNAs in plasma 67.

However, there are still many issues to be resolved before applying exosomal circRNA as a biomarker, such as preservation of specimens,cell source of exosomes and extraction of exosomes, etc.

Currently, the specimens are mostly stored in a refrigerator at -80°C and the amount of clinical testing samples is relatively large, so the preservation of samples requires prior planning. In addition, it's hard to trace the source of exosomes extracted from circulating body fluids. Earlier studies have confirmed that the cell origin of exosomes can be inferred by detecting specific cell markers on the surface of exosomes. Recently, researchers used ultra-sensitive flow cytometry to confirm the source of a single extracellular vesicle by staining different cell-specific markers through flow cytometry 68. Then they counted the proportion of exosomes from different cell sources in the blood, and initially explored a way to trace the cell source of exosomes.

Given large quantities of clinical samples, it is necessary to clarify how to extract exosomes quickly, accurately, and completely, so that exosomal circRNA can be put forward into the clinic. However, there is no unified standard for the method and quality control of exosome isolation and purification, which is also the dominant problem restricting the research of exosome diagnosis technology.

At present, the most common R & D program for clinical testing of exosomes is based on the technology of separation and purification of exosomes through clinical molecular testing. For example, Exosome Diagnositics has developed and introduced exosome kits for cancer detection based on blood and urine samples. Its technical concept is to separate and purify exosomes from patients' various body fluids, and perform physical characterization of exosomes, quantitative detection of particle content, and molecular detection of content. So far, many techniques have also been developed to separate exosomes from various samples of patient's urine and blood. These techniques mainly include the classic ultracentrifugation, magnetic bead seperation, gel exclusion, immunoaffinity capture, and lectin-induced exosome aggregation or precipitation. Some of these methods have been streamlined by commercial kits. In addition, many new instruments based on granularity or other inherent characteristics have been developed 69. At present, the exosomes isolated by the above methods can be directly applied to classic clinical molecular detection techniques at RNA and protein levels, and even batch detection can be achieved by using the high-throughput second-generation sequencing technology, microarray chips, and proteomics analysis technology.

## Summary and Outlook

At present, the diagnosis of some tumor -related diseases in clinical practice often requires histopathological confirmation of the diagnosis; however, samples of some deep tissue lesions are difficult to obtain and require invasive examination, which can cause damage. Therefore, non-invasive diagnostic methods are imperative to detect the diseases early.

As a newly discovered biological molecule, circRNA is specifically expressed at different times, tissues, and stages of the disease. Compared with the traditional method of monitoring circRNA in body fluids, exosomes are the detection vectors, and more circRNAs are screened as novel markers, which are expected to reflect tumor specificity more comprehensively and overcome the problems of tumor heterogeneity and circRNA degradation (Figure 3). With the development of liquid biopsy technology and the emergence of circulating tumor cell (CTC) detection technology, tumor cell-derived exo-circRNAs have also become the hotspot of tumor markers.

CircRNAs in fluid exosomes have been proved to owning a certain value in the diagnosis, efficacy judgment, and prognosis observation of cardiovascular diseases, nervous system diseases, endocrine diseases and autoimmune diseases 70. With further exploring different circRNA, circRNA in exosomes will likely have more clinical significance for the early diagnosis, treatment monitoring and prognosis assessment of tumors.

## Figures and Tables

**Table 1:** CircRNAs that can be used as tumor biomarkers

Cancer Category	CircRNAs	Test Samples	Trends	Ref
Hepatocellular Carcinoma	has_circ_0001445 has_circ_104075	plasma serum	Down Up	36 57
Gastric Cancer	has_circ_002059 has_circ_0000190 has_circ_0000745 has_circ_0000663 has_circ_0000181 has_circ_0000520 has_circ_0006848	serum serum plasma plasma serum plasma plasma	Down Down Down Down Down Down Down	71 37 72 6 73 14
Breast Cancer	has_circ_0001785	serum	Down	74
Lung Cancer	has_circ_0013958 circFARSA	plasma serum	Down Up	75 76
Pancreatic Cancer	circ-LDLRAD3 chr14:101402109-101464448 and chr14:52729603-52780244	plasma plasma	Up Up	77 78
Oral Squamous Cell Carcinoma	has_circ_0001874 and has_circ_0001971	saliva	Up	4

**Table 2:** Exosomal circRNA in body fluids that can be used as tumor biomarkers

Cancer Category	Exosomal CircRNAs	Test samples	Potential value	Ref
Pancreatic Cancer	1.circ-IARS 2.circPDE8A	serum	Early diagnosis and prognosis	56 36
Stage III Endometrial Cancer	has_circ_0109046 and has_circ_0002577	serum	Early diagnosis	57
Gastric Cancer	1.circ-RanGAP1 2.has_circ_0010522	serum	1.Preoperative diagnosis and prognosis monitoring 2.Condition judgment	58 61
Hepatocellular Carcinoma	1.circPTGR1 2.circRNA-100338	serum	1.Preoperative diagnosis and prognosis monitoring 2.Prognosis monitoring	59 60
Laryngeal Squamous Cell Carcinoma	circRASSF2	serum	Preoperative diagnosis	43
Lung Cancer	1.circFECR1 2.cricRNA-0056616	serum	1. Dynamic prediction of chemotherapy response 2. Detection of lymph node metastasis	62 63
Papillary Thyroid Cancer	circFNDC3B	serum	Early diagnosis and prognosis	8
Bladder Urothelial Carcinoma	circPRMT5	urine	Early diagnosis	5

## Abbreviations

circRNA: circular RNA
miRNA: microRNA
EIciRNA: exon-intron circRNA
ecRNA: exonic RNA
ciRNA: circular intronic RNA
tricRNA: tRNA intronic circular RNA
TSEN: tRNA splicing endonuclease
BHB: bulge-helix-bump
MBL: muscleblind-like
c-Myc: cellular-myelocytomatosis viral oncogene
TGF-β1: transforming growth factor-β1
ESRP1: epithelial splicing regulatory protein 1
GPRC: G protein coupled receptor, family C
CSCs: cancer stem cells
HCC: hepatocellular carcinoma
ROC: receiver operating characteristic
TNM: Tumor Node Metastasis
CA19-9: carbohydrate antigen 19-9
CEA: carcinoembryonic antigen
AUC-ROC: area under the receiver operating characteristic curve
CA72-4: carbohydrate-associated antigen 72-4
T cell: thymus dependent lymphocyte
HUVEC: human umbilical vein endothelial cell
VEGF: vascular endothelial growth factor
EMT: epithelial-mesenchymal transition
UCB: urothelial carcinoma of the bladder
MMP2: matrix metallopeptidase 2
MDR: multi-drug resistance
TMZ: temozolomide
MAPK: mitogen-activated protein kinase
RBP: RNA binding protein
PDAC: pancreatic ductal adenocarcinoma
PRDM16: positive regulatory domain containing 16
LSCC: laryngeal squamous cell carcinoma
PTC: papillary thyroid cancer
SCLC: small cell lung cancer
lncRNA: long non-coding RNA
CTC: circulating tumor cell
